# Severe bilateral visual loss as the first sign of IgA nephropathy

**DOI:** 10.5935/0004-2749.2021-0314

**Published:** 2023

**Authors:** Leonardo Provetti Cunha, Raphael Lucas Sampaio Defina, Rony Carlos Preti, Luciana Virgínia Ferreira Costa-Cunha, Leandro Cabral Zacharias, Kenzo Hokazono, Mário Luiz Ribeiro Monteiro

**Affiliations:** 1 Faculdade de Medicina, Universidade Federal de Juiz de Fora, Juiz de Fora, MG, Brazil; 2 Postgraduate Program, Faculdade de Medicina, Universidade de São Paulo, São Paulo, SP, Brazil; 3 Hospital de Olhos de Juiz de Fora, Juiz de Fora, MG, Brazil; 4 Universidade Federal do Paraná, Curitiba, PR, Brazil; 5 Faculdade de Medicina, Universidade de São Paulo, São Paulo, SP, Brazil

**Keywords:** Berger’s disease, Glomerulonephritis, IGA, Hypertensive retinopathy, Tomography, optical coherence, Macular edema, Hypertension, malignant, Systemic arterial hypertension, Doença de Berger, Nefropatia por IgA, Retinopatia hipertensiva, Tomografia de coerência óptica, Edema macular, Hipertensão maligna, Hipertensão arterial sistêmica

## Abstract

We describe a case of a 33-years-old woman who presents with severe acute bilateral visual loss secondary to massive exudative hypertensive maculopathy as the first sign of immunoglobulin A nephropathy. The patient’s ophthalmic examination showed bilateral cotton-wool spots, flame-shaped retinal hemorrhages, diffuse narrow arterioles, optic disk edema, and exudative maculopathy. Systemic workup demonstrated a systolic and diastolic blood pressure of 240 mmHg and 160 mmHg, respectively, proteinuria, and hematuria, suggesting kidney disease as the causative condition. A kidney biopsy confirmed immunoglobulin A nephropathy. She was treated with systemic corticosteroids, antihypertensive drugs, and a single bilateral intravitreal injection of aflibercept. There was a prompt resolution of macular edema and vision improvement. Our case draws attention to the fact that severe bilateral visual loss can be the first sign of severe hypertension. Secondary causes, such as immunoglobulin A nephropathy, should be ruled out.

## Abstract (pt)

Nosso objetivo é descrever uma paciente de 33 anos de idade, com perda visual bilateral grave por maculopatia hipertensiva exsudativa como o primeiro sinal da nefropatia por imunoglobulina A. A fundoscopia revelou a presença de manchas algodonosas, hemorragias em chama-de-vela, estreitamento arteriolar difuso, edema de disco óptico e maculopatia exsudativa bilateral. A pressão arterial sistólica foi de 240mmHg e a diastólica de 160 mmHg associado a proteinúria e hematúria, sugerindo a presença de doença renal. A biópsia renal confirmou a nefropatia por imunoglobulina A. A paciente foi tratada como corticoide sistêmico, drogas anti-hipertensivas e uma única dose intravítrea de Aflibercept em ambos os olhos. Houve rápida melhora do edema macular e da acuidade visual. Nosso caso chama a atenção para o fato de que a perda visual bilateral grave pode ser a primeira apresentação de uma doença hipertensiva sistêmica. Causas secundárias como a nefropatia por imunoglobulina A devem ser afastadas.

## INTRODUCTION

Systemic arterial hypertension can affect the eye in several ways, including damaging the optic nerve, retina, and choroid^(1)^. While the changes affecting primarily the retinal vasculature are generally subtle and asymptomatic in essential hypertension, the ocular findings in secondary hypertension can be striking, leading to severe visual loss. Secondary hypertension can occur in the setting of other conditions, such as pheochromocytoma, primary hyperaldosteronism, Cushing disease, and kidney disease^(2)^. Such conditions are more common in young patients and may present as bilateral visual loss due to exudative maculopathy^(1)^. Thus, the purpose of this study is to report a case of a young woman with severe bilateral visual loss due to hypertensive retinopathy from immunoglobulin (Ig) A nephropathy.

## CASE REPORT

**Figure 1:**
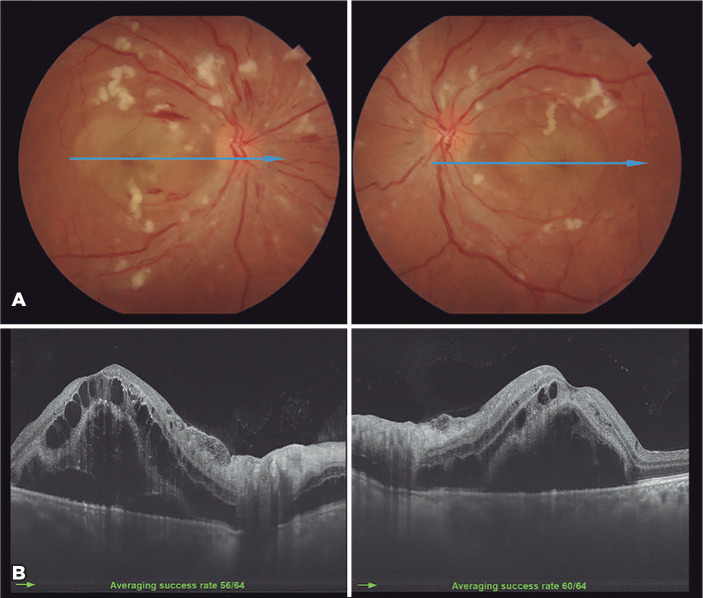
(A) A fundus retinography of both eyes shows bilateral cotton-wool spots, flame-shaped retinal hemorrhages, diffuse narrowed arterioles, mild optic disk edema, and exudative maculopathy. A blue horizontal line shows an optical coherence tomography (OCT) scanned area. (B) Swept-source OCT B-scans show bilateral macular edema with intraretinal cysts and a massive subretinal fluid in both eyes.

**Figure 2:**
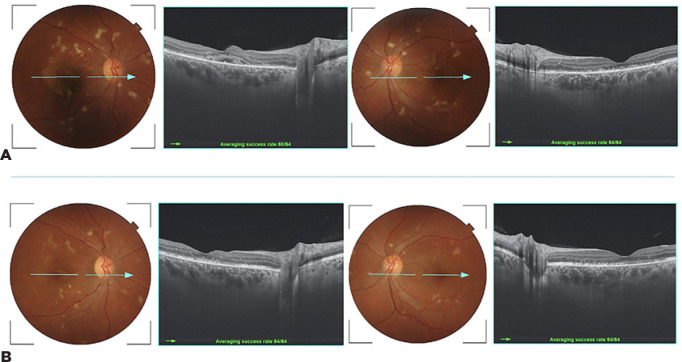
(A) Fundus retinography and swept-source OCT B-scans three weeks after the procedure show remarkable improvement in both eyes. (B) Note the additional absorption of subretinal fluid in the OD after 10 weeks.

A 33-year-old woman presented with sudden bilateral visual loss in both eyes (OU) over three weeks. She had a three-year history of uncontrolled systemic arterial hypertension. Best-corrected visual acuity (BCVA) was 20/400 in the right eye (OD) and 20/200 in the left eye (OS). Ocular motility and anterior segment eye examination were unremarkable. Pupils were equal in size with a normal reaction to light. The accommodation was intact. The fundus exam showed bilateral cotton-wool spots, flame-shaped retinal hemorrhages, diffusely narrowed arterioles, mild optic disc edema, and exudative maculopathy (Figure 1A). Swept-source optical coherence tomography (OCT) B-scans showed bilateral macular edema with intraretinal cysts and massive subretinal fluid in OU (Figure 1B). A systemic evaluation revealed a blood pressure of 240/160 mmHg. The patient was hospitalized for hypertension treatment and additional tests. The complete blood count was normal, and serum creatinine was 1.29 mg/dl (reference value (RF): 0.4 to 1.30 mg/dl). Urinalysis demonstrated 4+ proteins, 2+ hemoglobin, and numerous red blood cells (30 per field). Examination of 24-hour urine, in a volume of 1.800 ml, showed proteinuria of 2.592 mg/24 hours (RF <150mg /24 hours). The kidney biopsy showed a granular deposition of IgA and complement (C3) in immunofluorescence staining in an expanded mesangium with foci of segmental and necrotizing proliferative lesions, confirming the immunoglobulin (Ig) A nephropathy. Because of the massive subretinal fluid and severe visual loss, aflibercept 2 mg (0.05 ml) as an intravitreal injection was performed in OU. Three weeks after the procedure, visual acuity (VA) improved markedly to 20/25 in OD and 20/20 in OS. There was also a significant improvement of the exudative maculopathy (Figure 2 A and B). After 10 weeks, there was no sign of sub- or intraretinal fluid, and the BCVA was 20/20 in OU (Figure 2 C and D).

## DISCUSSION

IgA nephropathy is a nephritic syndrome characterized by the deposition of IgA immunocomplexes in glomeruli^(3)^. Clinical manifestation includes hematuria, proteinuria, and slowly progressive renal failure. The diagnosis is based on urinalysis and renal biopsy. Despite the 3-year history of uncontrolled hypertension, our case was asymptomatic. The patient was treated as for essential hypertension before. Due to acute visual loss and severe hypertensive retinopathy, a secondary hypertension hypothesis was raised.

Retinal abnormalities in severe hypertensive retinopathy include intraretinal transudate, multiple cotton-wool spots, superficial retinal hemorrhages, diffuse narrow arterioles, optic disc edema, and exudative maculopathy^(1)^. In our case, massive subretinal fluid was the main cause of visual loss, most likely due to an increased choroidal thickness, interstitial fluid accumulation, and an increase in hydrostatic pressure, leading to fluid movement across the retinal pigment epithelium into the subretinal space as a consequence of an external blood-retinal barrier breakdown^(4)^. On the other hand, accumulation of intraretinal exudative fluid can result from direct leakage through the retinal capillaries plexus due to the internal blood-retinal barrier breakdown^(4,5)^.

Ahn et al.^(5)^ investigated retinal and choroidal changes using OCT in patients with severe hypertension. They found that subretinal fluid height at baseline was significantly correlated with both baseline and final VA in patients with severe hypertension, suggesting that a greater subretinal fluid accumulation was related to a more significant persistent visual loss^(5)^. In this study, retinal and choroidal thickness started to decrease after blood pressure control and plateaued by the 1-month visit. However, time for anatomical improvement among patients showed significant variation, ranging between 2 to 36 months (average of 8.8 months). In some cases (16.7%), retinal abnormality persisted until the final visit^(5)^.

Therefore, the amount of subretinal fluid and its rate of reabsorption may be strictly related to the chances of visual recovery^(5)^. Treatment that may allow faster resorption of intra- and subretinal fluid is desirable to achieve more significant visual and anatomical improvement and minimize the possibility of a more substantial photoreceptors loss that could impair the final vision.

Widely used antivascular endothelial-derived growth factor (VEGF) drugs are recognized to reduce retinal vascular permeability, stabilize blood-retinal barriers, and promote subretinal fluid reabsorption.

Few previous studies reported the use of intravitreal bevacizumab injection in exudative hypertensive maculopathy^(6-9)^. In our case, we considered this therapeutic option as an adjunct to systemic hypertension control due to the severity of visual loss and massive anatomic changes. We were concerned with the possibility of permanent visual impairment related to photoreceptor loss^(4,5,10)^. Three weeks after aflibercept intravitreal injection, our patient achieved significant visual improvement and a striking resolution of the retinal changes.

Our case is interesting because the visual loss was the first sign that made the patient seek medical help. The presence of severe hypertensive retinopathy drew attention to the possibility of secondary hypertension, and hematuria and proteinuria signs pointed toward kidney disease.

Our study has some limitations since it is a single case report. Moreover, we should consider that hypertensive retinopathy may resolve only with systemic antihypertensive treatment. Nonetheless, we believe that anti-VEGF agents may speed up visual and anatomic recovery.

In conclusion, we have described a case of severe visual loss secondary to massive exudative hypertensive maculopathy. This condition should draw attention to secondary hypertension-related causes, such as IgA nephropathy. Also, aflibercept intravitreal injection may be a therapeutic option in selected cases combined with systemic antihypertensive treatment.
